# Maprang “*Bouea macrophylla* Griffith” seeds: proximate composition, HPLC fingerprint, and antioxidation, anticancer and antimicrobial properties of ethanolic seed extracts

**DOI:** 10.1016/j.heliyon.2019.e02052

**Published:** 2019-07-11

**Authors:** Nathupakorn Dechsupa, Jiraporn Kantapan, Montree Tungjai, Sorasak Intorasoot

**Affiliations:** a Department of Radiologic Technology, Faculty of Associated Medical Sciences, Chiang Mai University, 110 Intawaroros Rd., Sripoom, Chiang Mai, 50200, Thailand; b Department of Medical Technology, Faculty of Associated Medical Sciences, Chiang Mai University, 110 Intawaroros Rd., Sripoom, Chiang Mai, 50200, Thailand

**Keywords:** Food science, Natural product chemistry, *Bouea macrophylla* Griffith, Maprang, Antioxidant, Anticancer, Antimicrobial, HPLC

## Abstract

In this study, the Maprang (*Bouea macrophylla* Griffith) seeds of 3 Thai varieties of this plant were studied in terms of nutrition, phytochemicals, chemical antioxidants and the bioactivity of their extracts. Maprang seeds revealed high levels of carbohydrates, dietary fiber, energy, potassium, phosphorus, magnesium, and calcium. The Maprang seed extracts possessed a high polyphenolic content and exhibited antioxidant properties against DPPH˙, ABTS˙^+^, and ferric reduction. Additionally, 18-compounds were charaterized by RP-HPLC-DAD with two being recognized as gallic acid and ellagic acid and 16-unknown gallotannins. The HPLC fingerprint was composed of 4 major compounds. The extract showed active growth inhibition against leukemia, lung cancer cell lines and for 15 strains of bacteria. It is known to be particularly effective in drug resistant cells. Our results indicated that maprang seeds are a new natural source of nutrition, minerals and phytochemicals that may be applicable for use as a food supplement and as an effective drug in the treatment of certain diseases.

## 1 Introduction

Epidemiological studies have pointed out that regular consumption of fruits and vegetables is associated with a lower risk of non-communicable diseases including cancer, diabetes and cardiovascular diseases [1, 2]. The health-promoting potential of these foods may be due to the phytochemical bioactive compounds that are present in these plants. Polyphenolics are the most abundant bioactive compounds present in fruits and vegetables, and are known for their medicinal properties. They exhibit a wide range of pharmacological advantages due to their antioxidant, anticancer, antimicrobial, and anti-inflammatory properties [3, 4]. They contain a mechanism related to these properties that involves the reduction of oxidative stress by scavenging free radicals [5]. There are many mothods that are used to recover phytochemicals from plants, such as Soxhlet extraction, maceration, pressurized fluid extraction, supercritical fluid extraction, subcritical water extraction and ultrasound-assisted extraction. Solvent extractions are the most commonly used method for plant extraction due to their ease of use, efficacy and wide applicability. It is generally known that the yield of chemical extraction depends on the type of solvents that can be associated with varying polarities, extraction times, temperatures, the ratio of solvent to solid, the part of the plant used, as well as the sample preparation process. The most suitable solvent extractions for plant phenolics are aqueous mixtures containing ethanol, methanol, acetone, and ethyl acetate. Ethanol has been recognized as an effective solvent for polyphenol extraction and is notably safe for human consumption [6, 7].

**Fig. 1:**
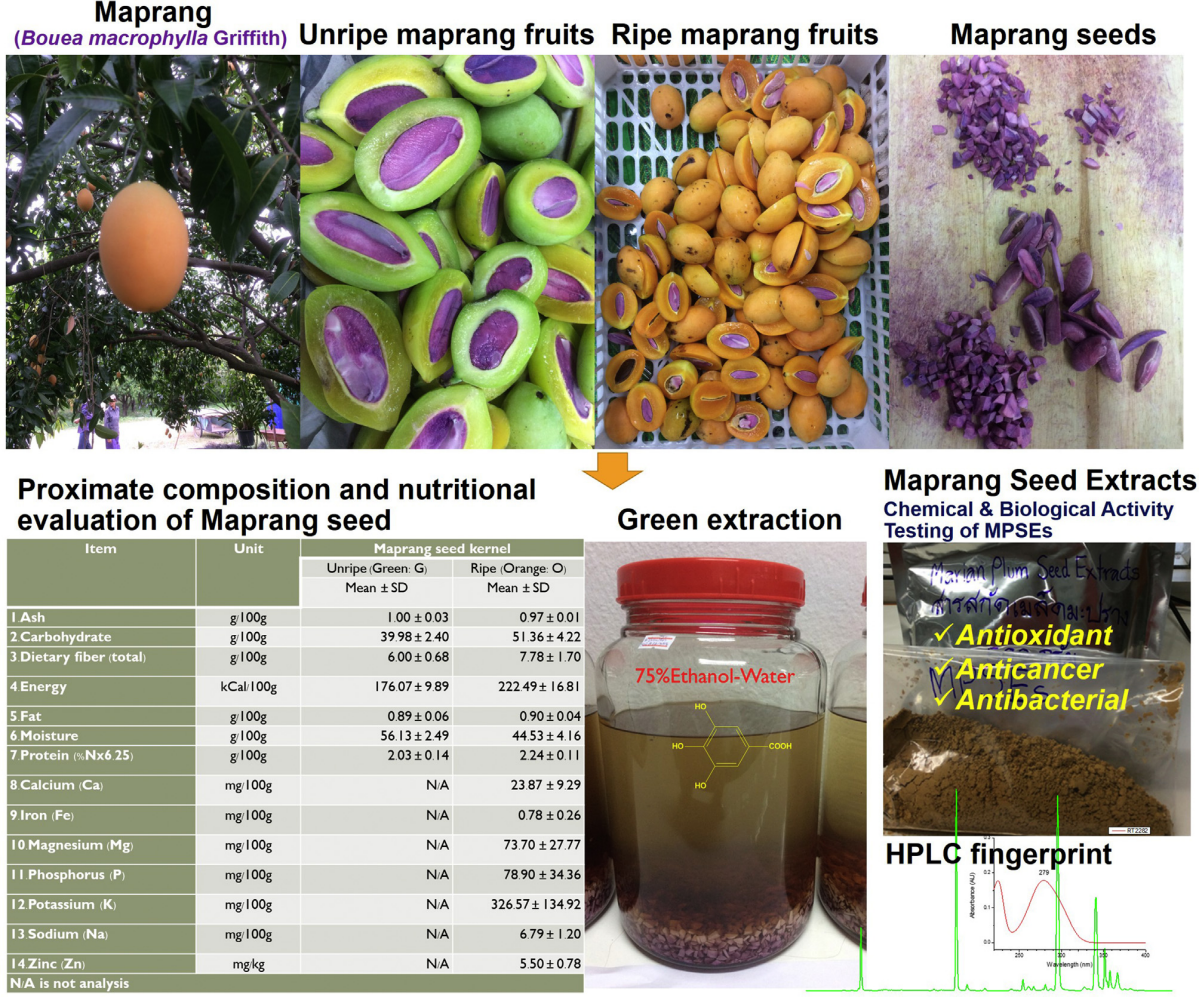
Rerearch diagram of *Bouea macrophylla* Griffith.

Marian plums, plum mangos, gandaria or maprang (*Bouea macrophylla* Griffith) (Fig. 1) is a tropical fruit that is widely grown throughout South East Asia, and particularly in the countries of Thailand, the Philippines, Malaysia, and Indonesia. The Marian plum, known as “Maprang” in Thailand, belongs to the same family as mangos (Anacardiaceae*)*, but the taste is notably different. Maprang tastes a bit like a mango if one eats the fruit with the rind, and a bit like a plum due to its pulp-like texture. However, the rind of the fruit is also edible. There are three varieties of maprang found in Thailand that are clasified according to taste [8]. The acidic variety is known as “sour maprang or maprang prieyo”. The sweet variety is the most popular sort of maprang, and is widespread throughout Thailand. It is called “maprang wan”. The last variety is called “mayong or mayong chid”. This variety is similar to the sweet maprang, but the ripened fruit can taste bitter. These fruits are commonly described as tasting sweet with an acidic flavor. Currently, maprang has been rapidly gaining popularity in Thailand. This tangy sweet fruit has a beautiful yellow-orange coloration when fully ripe, and is also high in vitamin C and beta-carotene [9]. Normally, the maprang fruit is either consumed fresh or pickled. Regionally, this fruit can be made into a compote or used in sambal (Indonesia), and rojak (Malaysia). The young leaves of the Maprang tree are also edible and they can be added to salads or served alongside vegetables, usually with chilies and shrimp paste. The Maprang seed is bitter in taste, but is also edible. These seeds represent about 60–150 g/kg (6–15%w/w) of the fruit, but they produce a very bitter taste. Typically, they are discarded during processing or consumption, yet the by-products of this plant may be an exploitable source of natural phenolic as an antioxidant.

A growing body of evidence suggests that fruit by-products (seeds and peels) are potentially rich sources of natural antioxidants and other bioactive compounds [10, 11]. These sources of bioactive compounds are inexpensive to obtain and are readily available for use. Furthermore, it would be beneficial to improve complete utilization of the seeds, which are typically discarded as waste. Various fruit seeds (mango, longan, litchi, tamarind, and rambutan etc.) have been reported to contain potential medicinal properties that can be used as antioxidant, anti-inflammatory, or antibacterial agents. They also display potential as being an anticancer ingredient [11, 12, 13, 14, 15]. Maprang fruit is one of Thailand's leading economic fruits, but there have not yet been any scientific studies that have reported on the nutritional value of its seeds, or even the medicinal properties of maprang seed extracts when compared to other fruits. Thus, the objective of this study is to report on the proximate analysis of maprang seeds in the unripe and ripened fruits, and to identify the polyphenol content by RP-HPLC profiling to determine total phenolic content and to identify any antioxidant, anticancer, or antimicrobial properties that are demonstrated (Fig. 1). This in-depth study may help to promote the cultivation of maprang trees in Thailand for economic benefit, but our main purpose is to find novel natural sources of antioxidants that are obtained from fruit by-products and that can be useful in health promotion beyond any commercial marketability of the fruit itself. Moreover, the transformation of this waste into healthful active ingredients without any harmful effects on food safety will be an economically favorable way of managing these agricultural waste products that are often expensive to dispose of properly.

## 2 Materials and methods

### 2.1 Reagents and standards

Type I water was purified with PURELAB Option-Q system (ELGA, England). The standard compounds included gallic acid (GA), caffeine (CF), (+)-catechin hydrate (C), (-)-epicatechin (EC), (-)-epicatechin gallate (ECG), (-)-epigallocatechin gallate (EGCG), (-)-gallocatechin (GC), (-)-gallocatechin gallate (GCG), tannic acid (Tannins), ellagic acid (EA), *trans*-ferulic acid (FA), pyrocatechol (PyC), protocatechuic acid (PA), vanillic acid (VA), resveratrol (RV), kaempferol (KF), quercetin (QT), apigenin (AN), delphinidin chloride, pelargonidin chloride, procyanidin B1, cyanidin chloride, malvin chloride, and ascorbic acid (Vit C), which were all purchased from Sigma-Aldrich. Additionally, Kesselguhr was also purchased from Sigma-Aldrich (Singapore).

### 2.2 Plant materials and extraction

Fifty kilograms of maprang fruit, both the unripe (more than 50 days after anthesis; Green, G) and the ripened (Orange, O) specimens in three varieties including maprang wan (sweet maprang), mayong chid, and maprang prieyo (sour maprang) were harvested during the period of March–April 2015 from the Marian Plum Plantation located in Nakhon Nayok Province (latitude and longitude: 14˚12′N and 101˚13′E). The fruit was triple-washed with tap water and transverse sectioned. Seeds were removed, weighed, and minced. Seeds were then dried with hot air at 60 °C for further extraction. Subsequently, 350 g of minced seeds were immersed in 3.5 L of various hydro-ethanolic systems (25%, 50%, 75%, and 95%EtOH) in order to macerate them for 7 days with daily shaking. The extraction solutions were filtered through Kesselguhr and were dried by evaporation at 40 °C at an approximate rotation speed of 200 rpm using a rotary evaporator (Buchi Rotavapor R-210, Switzerland). The crude extract of the maprang seeds was collected and stored at room temperature in a desiccator for further study. The extraction yield was then determined.

### 2.3 Proximate analysis of maprang seeds

Fifty kilograms of maprang fruit were transported to the Central Laboratory (Thailand) Company Limited, Chiangmai Branch and were certified (accreditation number 1079/48) by the Bureau of Laboratory Quality Standards, Ministry of Public Health, Thailand for further proximate and mineral analysis. The fresh maprang seeds were analyzed for their energy levels, moisture, protein, lipid, starch, dietary fiber, ash, and mineral content. Briefly, carbohydrate and energy contents were determined according to a method of food analysis based on a contendum of a 1^st^ edition source that was published in Thailand in 2003. Moisture content was determined according to AOAC (2012) 925.10 and 950.46 methods. Protein contents were determined by AOAC (2012) 991.20 which is commonly referred to as the Kjeldhal Method. Lipid content was determined by AOAC (2012) 9948.15 method. Ash was determined according to AOAC (2012) 923.03 and 920.153 methods. The dietary fiber content was determined by the AOAC (2010) 985.29 method. Minerals were determined by using an ICP-OES technique based on the in-house method of TE–CH–170 listed in the 18^th^ edition of 2005, Ch.50 (984.27) and Ch.9 (999.10). Only zinc could be determined by the ICP-MS technique, which was employed to determine mineral content using an in-house method based on AOAC 2005.999.10.

### 2.4 Phytochemical analysis of MPSEs by HPLC

The stock solution of MPSEs was freshly prepared at 1 mg/mL volume in purified water and was filtrated with a 0.45 μm syringe filter (Whatman's). HPLC profiles of MPSEs were performed using a Shimadzu LC-20AD Prominence Liquid Chromatograph system equipped with a SPD-M20A Prominence Diode Array Detector and with a DGU-20A3 Prominence Degasser (Shimadzu, Japan). The wavelength scanning was done between 190 and 800 nm, while any wavelength occurring at 270 nm was carefully monitored. Notably, 20 μL of each extract (300 μg/mL) was injected into the ZORBAX Eclipse Plus C18 Analytical column (250 × 4.6 mm i.d., 5 μm particle) with an Eclipse Plus-C18 Analytical Guard Column (12.5 × 4.6 mm i.d., 5 μm particle. Successful separation of MPSEs phytochemicals was accomplished using the following mobile phase; mobile A: 0.1% aqueous trifluoroacetic acid and mobile B: 100% acetonitrile. The time program for gradient elution was 0–5 minutes, 5% B; 7–12 minutes, 10% B; 14–19 minutes, 15% B; 21–26 minutes, 20% B; 30–35 minutes, 25% B; 37–45 minutes, 30% B; 50–55 minutes, and 100%B; 60–65 minutes, 5% B.

### 2.5 Determination of total phenolic acid by Folin-Ciocalteu assay

Maprang seed extracts were prepared at a concentration of 1 mg/mL in water. 125 μL of each MPSE solution was mixed together with 500 μL of water and 125 μL of 2M-Folin-Ciocalteu (Sigma) in a 24-well plate. The reaction was allowed to progress for 6 minutes before 1.25 mL of 7% Na_2_CO_3_ was added to a total volume of 3 mL with water. The solution was mixed well to keep the reaction progressing at room temperature in the dark for 90 minutes. After that, the absorbance at 760 nm of each reaction was measured using a UV-Vis Spectrophotometer (Agilent 8453, USA). The total phenolic content was determined from the standard curve of gallic acid and expressed as a gallic acid equivalent, and listed as mg GAE/mg of maprang [16].

### 2.6 Determination of antioxidation activity by FRAP assay

Fresh solutions were prepared of: (1) 300 mM-Acetate buffer (pH = 3.6), (2) 10 mM-TPTZ (2, 4, 6 -tri [2-pyridyl]-s-triazine) by dissolving 0.031 g TPTZ in 10 mL of 40 mM-HCl at 50 °C, and (3) 20 mM-FeCl_3_. Next, a FRAP solution was prepared by mixing all three of the solutions by means of a volume ratio between solutions 1, 2, and 3 in order to obtain a 10:1:1 ratio; with a final volume of 10 mL. Additionally, 3000 μL of FRAP solution was mixed together with 100 μL of 1 mg/mL-MPSEs to keep the reaction stable at room temperature in the dark for 8 minutes. Afterward, the absorbance at 593 nm for each reaction was measured using a UV-Vis spectrophotometer. Ferrous sulfate (FeSO_4_.7H_2_O) was used as a standard anti-oxidation agent for the FRAP [16]. The antioxidant activity of the test compound was expressed as a Fe^2+^ equivalent, i.e. μg Fe^2+^E/μg of maprang.

### 2.7 Determination of antioxidation activity by TEAC assay

This was done by preparing 2.45 mM-potassium persulfate (K_2_S_2_O_8_) into 7 mM-ABTS (2,2′-azino-bis(3-ethylbenzthiazoline-6-sulphonic acid)). This solution was then incubated at room temperature in the dark for 12–16 hours to produce ABTS˙^+^ radicals. Afterward, these free radicals were diluted using ethanol to give an absorbance of 752 nm equalling 0.7 ± 0.02 AU as ABTS˙^+^ stock solution [16]. The reaction of 1 mg/mL-maprang (10 μL) and ABTS˙^+^ stock solution (990 μL) ocurred at room temperature in the dark for 4 minutes, and an absorbance of 752 nm was measured by UV-Vis spectrophotometer. Trolox (0, 50.058, 100.116, 150.174, 200.232, and 250.29 μg/mL) was used as a standard to scavenge ABTS˙^+^. The scavenging activity at 50% (IC_50_) of the test compound was expressed as Trolox equivalent, i.e. μg TE/μg of the maprang extract.

### 2.8 Determination of scavenging radical activity by DPPH assay

A free radical solution of 0.2 mM-DPPH (di-(phenyl)-(2,4,6-trinitrophenyl) iminoazanium) was prepared in ethanol. A solution of 0.5 mL of MPSEs was mixed with 2.5 mL of 0.2 mM-DPPH to obtain a final concentration of MPSEs at 0, 12.5, 25, 50, and 100 μg/mL. Time was allowed for the reaction to proceed at room temperature in the dark for 30 minutes. Afterward, the absorbance at 517 nm of each reaction was measured using a UV-Vis spectrophotometer [16]. The percentage of DPPH-scavenging activity (%IC) by test compound was evaluated using the following Eq. (1):

(1) %IC = [1- (A-B)/A_0_] x 100

where A = absorbance of sample, B = absorbance of 0.5 mL of extracts +2.5 mL of ethanol, and A_0_ = absorbance of control. The scavenging radical activity at 50% (IC_50_) of MPSEs was evaluated from the plot of %IC and the concentration relationship. Vitamin C was used as a positive control.

### 2.9 Anticancer properties

All green maprang seed extracts were obtained by various extraction systems and were selected to be tested for anticancer properties. Drug sensitive and drug resistant cancer cell lines of erythromyelogenous lukemic cells (K562 and K562/*adr*) and small cell lung carcinoma cells (GLC4 and GLC4/*adr*) were used in this study. They were grown in a RPMI1640 medium combined with 10% fetal bovine serum and 1% penicillin-streptomycin at 37 °C with 5% CO_2_ in a humidified CO_2_-incubator. The concentrations of MPSEs were prepared at 10 mg/mL in water. Various concentrations of MPSEs (0, 10, 20, 40, 50, 60, 80, 100, 110, 120, 130, and 150 μg/mL) were incubated with cancer cells that had a beginning quantity at 1 × 10^4^ cells in 1 mL of completed RPMI1640 medium. They were then placed in a humidified CO_2_-incubator for 72 hours. Afterward, the number of cells were counted by flow cytometer (Beckman Coulter Epics XL), and the percentage of inhibition cancer cell growth (%IC) was calculated using the following Eq. (2):

(2) %IC = [1- (N_MPSEs_)/(N_C_)] x 100

N_C_ and N_MPSEs_ were determined by the number of cells present at 72 hours after initiating seeding time for the control and the MPSEs treatment groups, respectively. The anticancer activity was determined by the concentration of MPSEs that had inhibited cancer cell growth by 50% (IC_50_). In addition, the resistance factor (RF) between drug resistant and drug sensitive cell lines was determined by the ratio of IC_50_ (of resistant cell)/IC_50_ (of sensitive cell).

### 2.10 Antimicrobial activity

#### 2.10.1 Microbial strains

A total of 15 bacterial strains consisting of *Staphylococcus aureus* ATCC 25923, methicillin-resistant *Staphylococcus aureus* ATCC 43300, *Enterococus faecalis*, vancomycin-resistant *Enterococcus faecalis*, *Escherichia coli* ATCC 25922, extended-spectrum beta-lactamase producing *E. coli*, *Proteus mirabilis*, *Salmonella enteritidis*, *Shigella boydii*, *Vibrio cholerae*, *Vibrio parahemolyticus*, *Klebsiella pneumoniae*, *Enterobacter aerogenes*, *Pseudumonas aeruginosa*, and *Acinetobecter buamannii* were examined. One yeast strain, *Candida albicans* ATCC 90028, was also examined in this study. All bacteria were subcultured in Mueller-Hinton agar (MHA) at 37 °C with shaking at 200 rpm for 18–24 hours, while the yeast cells were grown in Sabouraud dextrose agar (SDA) at 37 °C for 24–48 hours.

### 2.10.2 Preparation of maprang seed extracts for antimicrobial activity testing

Maparang seed extracts (100 mg) of both the unripe (MYCG95) and ripe (MYCO95) fruit were prepared by putting them into a 50 mg/mL solution using 5% DMSO in order to dilute the specimens to a desired concentration of 5 mg/mL using Mueller-Hinton broth (MHB). Seed extracts were filtered with a 0.22 μm syringe filter, aliquoted, and stored at -70 °C until it was time for analysis.

### 2.10.3 Agar well diffusion method for antimicrobial activity determination of seed extracts

Prior to determination of antimicrobial properites, an agar well diffusion assay was performed to evaluate the antimicrobial aspects. *Escherichia coli* ATCC 25922 was selected as a microorganism candidate in this study. Briefly, an overnight culture of bacteria was subcultured into MHB and incubated at 37 °C with shaking at 200 rpm until the OD600 reached 0.6–0.8. The bacterial suspension was adjusted to approximately 1 × 10^8^ CFU/mL with McFarland standard No. 0.5 and the suspension was spread over the surface of MHA plates using a sterile cotton swab. The media was punched aseptically with a sterile cork borer and 100 μL of 5 mg/mL seed extracts were individually added. Gentamicin (40 μg/mL) was introduced into the well as a positive antibiotic control. The plate was then incubated at 37 °C for 18–24 hours, and the inhibition zone was measured in millimeters (mm). The experiments were performed independently in triplicate and the mean value was calculated.

### 2.10.4 Broth microdilution assay for antimicrobial activity determination of seed extracts

The broth microdilution method was performed according to the Clinical and Laboratory Standard Institute (CLSI) standard procedure. The procedures followed M07-A9 guidelines for bacteria (2012) and M27-A3 for yeast (2008). Briefly, the bacteria were cultured in MHB and incubated at 37 °C with agitation at 200 rpm overnight. The culture was adjusted to approximately 1 × 10^8^ CFU/mL with McFarland standard turbidity No. 0.5. Subsequently, 50 μL of diluted bacteria was mixed with 50 μL of 2-fold serially diluted seed extract. The final bacterial inoculum was recorded at approximately 5 × 10^5^ CFU/mL. Gentamicin and 5% DMSO were used as the antibiotic and solvent control, respectively. Plates were incubated at 37 °C for 20 hours. The minimum inhibitory concentration (MIC) was observed after the addition of 0.2 mg/mL p-iodonitrotetrazolium chloride (INT, BioChemica, Germany) in all wells and was further incubated at 37 °C for 30 minutes. The color change from a clear solution to pinkish-red formazan indicated bacterial growth. The MIC interpretation was identified by the dilution of the seed extract that displayed no visible pink color [17]. The experiments were separately performed in triplicate and the mean and standard deviations (SD) of MIC and the minimum bactericidal concentration (MBC)values were both calculated. Next, the minimum concentration of the seed extract that had killed 99.9% of the tested microbial population was determined. A colony count was performed individually to calculate the number of initiating bacterial cells used.

In order to determine the level of antifungal activity, an isolated colony of *C. albicans* was cultured in SDA and incubated at 37 °C overnight. The culture was adjusted to the No. 0.5 McFarland standard turbidity (1 × 10^6^ CFU/mL) using RPMI 1640-MOPS medium, followed by dilution 1:100 and 1:20 with fresh medium. One hundred microlitres of yeast suspension (approximately 5.0×10^2^–2.5 × 10^3^ CFU/mL) was mixed with 100 μL of 2-fold serially diluted seed extract. Fluconazole and 5% DMSO were used as the antifungal drug and solvent control, respectively. The microtiter plate was incubated at 37 °C for 48 hours. These experiments were separately performed in triplicate and both the mean and SD of the MIC, and the minimum fungicidal concentration (MFC) value, were calculated.

### 2.11 Statistical analysis

The data are presented as mean ± standard deviation (SD) of triplicate measurements. The data were analyzed by one-way analysis of variance (ANOVA), followed by Tukey's test to detect significanct differences among means of each factor with the level of significance being *p* < 0.05. Statistical analysis was performed using OriginPro2018 software.

## 3 Results and discussion

### 3.1 Extraction yield of maprang seed extracts using ethanolic solvent system

Maprang seed kernels were the portion of the fruit used to extract maprang flavonoids and for the purposes of further studying their chemical, antioxidant, and biological properties. The percentage (%w/w) of fresh seed kernels to fresh maprang fruit was 14.04% (MPWG), 12.82% (MPPG), and 6.49% (MYCG) for unripe seeds. For ripe seeds, it was 14.71% (MPWO), 13.62% (MPPO), and 5.7% (MYCO). We found a slight increase in the number of seed kernels from the unripe to the ripe fruit being about 0.7% in maprang wan and 0.8% in maprang prieyo, but the opposite tendency was found in mayong chid with a 0.7% decrease being observed. In comparison to mango fruit where the seed kernel represents approximately 20% of the whole fruit [18], this percentage was higher in the mango fruit than in the maprang fruit by about 5–15% depending on the variety. The extraction yields of the maprang seed extracts (MPSEs) obtained from three maprang varities using various percentages of ethanolic solvents (25%, 50%, 75%, and 95%EtOH) are indicated in Table 1. In our extraction systems, we obtained the extraction yield that varied in a range of 8–16%; with a minimal yield of 8.32% for MYCO having 25% EtOH. A maximum yield was found at 16.17% in MYCG with 50%EtOH. Unripe fruit revealed a higher extraction yield than ripe fruit in all extraction systems, and this was the case for all three maprang fruit varieties. For example, with the determination solvent system at 75%EtOH, extraction yields of MPWG (12.60%) > MPWO (11.72%), MYCG (12.67%) > MYCO (10.08%), and MPPG (12.40%) > MPPO (11.92%) were recorded. Dorta et al reported the extraction yield of mango seed kernels by using 50% aqueous acetone with the microwave-assisted extraction method at 50 °C depending on the seed drying process. It was found that the extraction yield was 12% in the freeze-drying process and 7.7% in the oven-drying process at 70 °C [19]. Under our extraction conditions using hot air oven-drying at 60 °C and maceration at room temperature for 7 days, the extraction yields were higher than the results obtaind by the oven-drying process used by Dorta et al. However, Nakpanich et al reported that the extraction yield of defatted mango seed kernels was obtained by 16% of both the raw and ripe mango fruit. Mango seed kernels were defatted before being dried in a hot air oven at 60 °C for 24 h and macerated again in 95% ethanol for 48 h, 3 cycles [20]. Maisuthisakul extracted phytochemicals from mango seed kernels using various extraction methods by shaking (4.5h) in 95% ethanol, refluxing (3h) with 95% ethanol and with 1.2 M HCl in ethanol. The highest extraction yield was obtained by refluxing with ethanol (11.90%) and followed by acid hydrolysis refluxing (10.75%) and shaking (3.31%), respectively [21]. By using the soaking method of supercritical fluid extraction (pressure of 42 MPa, 72 °C, CO_2_ flow rate of 3.4 mL/min), maprang seed fat was obtained by 11% yield [22]. Indeed, the extraction yield of phytochemicals in addition to the part being used depends upon various factors including extraction solvent, method and time [23, 24, 25]. In this study, it was indicated that the seed kernel extraction yield of the maprang fruit is dependent upon the variety, the maturity of the fruit and the percentage of the ethanol in the extraction solvent. All together, it can be summarized that the extraction yield of the seed kernels obtained from the mango and maprang fruit was found at about 5% and up to 16%.

**Table 1:** Extraction yield and antioxidant activity of MPSEs.

Ethanolic extraction system	MPSEs	Extraction yield (%) DW		Total phenolic acid mg GAE/mg MPSEs		Antioxidation activity μg TE/μg MPSEs		Antioxidation activity μg Fe^2+^E/μg MPSEs		Radical scavenging activity IC_50_ μg/mL	
		Unripee	Ripe	Folin–Ciocalteu assay		TEAC assay		FRAP assay		DPPH assay	

%Ethanol	Maprang wan	Green	Orange	Green	Orange	Green	Orange	Green	Orange	Green	Orange
95	MPW(G/O)95	12.32	9.40	0.39 ± 0.01 (c, A, b)	0.43 ± 0.01 (b, B, c)	2.01 ± 0.47 (a, A, a)	1.38 ± 0.44 (c, A, a)	103.55 ± 16.73 (a, A, ab)	121.67 ± 19.46 (ab, A, a)	31.79 ± 12.66 (a, A, a)	28.80 ± 10.54 (a, A, a)
75	MPW(G/O)75	12.60	11.72	0.49 ± 0.01 (b, A, b)	0.50 ± 0.00 (a, A, a)	1.52 ± 0.39 (a, A, a)	3.17 ± 0.63 (a, B, a)	109.19 ± 15.42 (a, A, a)	139.86 ± 18.36 (a, A, a)	28.74 ± 3.33 (a, A, a)	25.07 ± 11.09 (a, A, a)
50	MPW(G/O)50	15.68	11.48	0.55 ± 0.02 (a, A, b)	0.41 ± 0.00 (b, B, a)	1.84 ± 0.1 (a, A, a)	1.96 ± 0.55 (ab, A, a)	90.91 ± 7.80 (a, A, a)	89.54 ± 11.67 (b, A, a)	29.38 ± 4.36 (a, A, a)	34.41 ± 6.42 (a, A, a)
25	MPW(G/O)25	12.36	11.28	0.42 ± 0.04 (c, A, b)	0.34 ± 0.01 (c, B, c)	1.89 ± 0.70 (a, A, a)	1.34 ± 0.69 (bc, A, a)	98.68 ± 4.77 (a, A, a)	80.81 ± 12.56 (a, A, c)	34.77 ± 10.09 (a, A, a)	44.35 ± 17.66 (a, A, a)
%Ethanol	Maprang prieyo	Green	Orange	Green	Orange	Green	Orange	Green	Orange	Green	Orange
95	MPP(G/O)95	12.20	9.04	0.42 ± 0.01 (c, A, b)	0.44 ± 0.00 (a, B, b)	1.47 ± 0.73 (a, A, a)	2.05 ± 0.41 (a, A, a)	79.66 ± 7.91 (b, A, b)	93.73 ± 19.39 (a, A, a)	34.98 ± 5.21 (a, A, a)	32.85 ± 6.73 (a, A, a)
75	MPP(G/O)75	12.40	11.92	0.45 ± 0.01 (b, A, c)	0.40 ± 0.00 (b, A, c)	1.60 ± 0.30 (a, A, a)	1.84 ± 0.29 (a, A, a)	87.18 ± 8.08 (b, A, a)	108.30 ± 23.98 (a, A, a)	28.62 ± 7.28 (a, A, a)	32.57 ± 10.99 (a, A, a)
50	MPP(G/O)50	12.20	11.60	0.45 ± 0.01 (b, A, c)	0.44 ± 0.02 (a, B, a)	1.80 ± 0.60 (a, A, a)	1.54 ± 0.65 (a, A, a)	107.49 ± 14.77 (b, A, a)	107.64 ± 19.59 (a, A, a)	28.46 ± 7.08 (a, A, a)	31.57 ± 10.10 (a, A, a)
25	MPP(G/O)25	11.60	11.08	0.60 ± 0.02 (a, A, a)	0.39 ± 0.01 (b, B, b)	1.89 ± 0.72 (a, A, a)	1.49 ± 1.02 (a, A, a)	116.46 ± 17.93 (a, A, a)	86.61 ± 15.42 (a, A, bc)	24.84 ± 4.85 (a, A, a)	46.34 ± 22.64 (a, A, a)
%Ethanol	Mayong chid	Green	Orange	Green	Orange	Green	Orange	Green	Orange	Green	Orange
95	MYC(G/O)95	11.22	9.96	0.77 ± 0.02 (b, A, a)	0.47 ± 0.00 (a, B, a)	2.21 ± 0.17 (a, A, a)	1.72 ± 0.48 (a, A, a)	114.98 ± 7.33 (ab, A, a)	94.82 ± 18.41 (a, A, a)	20.87 ± 1.90 (a, A, a)	31.14 ± 6.21 (a, A, a)
75	MYC(G/O)75	12.67	10.08	0.75 ± 0.01 (b, A, a)	0.42 ± 0.00 (b, B, b)	1.68 ± 0.40 (a, A, a)	1.79 ± 0.76 (a, A, a)	93.65 ± 4.97 (c,A, a)	118.79 ± 18.91 (a, A, a)	27.68 ± 5.91 (a, A, a)	28.59 ± 11.55 (a, A, a)
50	MYC(G/O)50	16.17	11.48	0.83 ± 0.03 (a, A, a)	0.40 ± 0.01 (c, B, a)	1.95 ± 0.25 (a, A, a)	1.56 ± 0.16 (a, A, a)	109.59 ± 6.48 (b, A, a)	93.27 ± 14.54 (a, A, a)	24.13 ± 2.95 (a, A, a)	36.40 ± 8.09 (a, A, a)
25	MYC(G/O)25	15.14	8.32	0.48 ± 0.01 (c, A, b)	0.41 ± 0.00 (bc, B, a)	2.23 ± 0.38 (a, A, a)	1.63 ± 0.39 (a, A, a)	124.88 ± 2.61 (a, A, a)	119.56 ± 14.14 (a, A, ab)	28.96 ± 7.47 (a, A, a)	33.06 ± 13.76 (a, A, a)
	**Vit C**									13.80 ± 2.26^∗^	

MPSEs, Maprang seed extracts; DW, Dry weight; G, Green (unripe fruit); O, Orange (ripe fruit); MPW, Maprang wan; MPP, Maprang prieyo; and MYC, Mayong chid; Data presented as mean ± standard deviation (n = 3); Statistical analysis was performed within columns for each variety as a function of %Ethanol (first lower case letter) and for three varieties at each %Ethanol (second lower case letter) and within rows (capital letter) between unripe and ripe fruits. Mean values sharing the same letter are not significantly different from each other (Tukey's test, *p* < 0.05).

* significant (*p* < 0.05) differences of all means within MPSEs.

### 3.2 Total phenolic content of maprang (*Bouea macrophylla* Griffith) seed kernel extracts

This is the first time that the total phenolic content (TPC) of maprang seed kernel extracts is being reported using various percentages of ethanolic solvent extraction, as is indicated in Table 1. The TPC values in Table 1 were varied between the unripe (green) and ripe (orange) maprang seed kernel extracts, and were also different among the three varieties: maprang wan (MPW), maprang prieyo (MPP), and mayong chid (MYC). These values were expressed as milligram of gallic acid equivalent weight per milligram of MPSEs dried weight ranging from 0.39 to 0.83 and 0.34–0.50 mg GAE/mg MPSEs for the unripe and ripe fruit, respectively. As is shown in Table 1, unripe maprang seed extracts exhibited the highest TPC values. Among the three varieties of unripe maprang seed extracts, TPC was the highest in MYCG (0.48–0.83 mg GAE/mg MPSEs), followed by MPPG (0.42–0.60 mg GAE/mg MPSEs), and MPWG (0.39–0.55 mg GAE/mg MPSEs). As for the ripe maprang seed kernel extracts, TPC values were lower than that of the unripe maprang seed extracts, and their values were not significantly different among these three varieties (*p* < 0.05). The TPC values obtainedof the *Bouea macrophylla* Griffith seed extract were obtained based on the TPC of 0.689 mg GAE/mg SEs; using hot water extraction results that were provided by Zainah et al [26]. The TPC of the maprang seed extracts was found to be higher than that of the mango (*Mangifera indica* L.) seed kernel extracts at 0.082–0.744 mg GAE/mg MSKEs; these findings have been well documented by Torres-León et al [18]. In addition, the TPC of the maprang seed extracts were greater than those of over 11 other fruit seed extracts ranging from 0.002-0.06 mg GAE/mg SEs. These others included *Amygdalus persica* Linn, *Annona squamosa* L., *Citrullus lanatus* (Thunb), Matsum and Nakai, *Dimocarpus longgana* Lour, *Durio zibethinus* L., *Eriobotrya japonica* (Thunb.) Lindl, *Litchi chinensis* Sonn, *Prunus salicina* Lindl, *Prunus salicina* Lindl (san hua), *Vitis vinifera* Linn (red), and *Vitis vinifera* Linn (black) [27]. *Bouea macrophylla* Griffith is a rich source of polyphenolic compounds that can be extracted from many parts of the plant including the seed kernels, leaves, cortex, and fruit. Rajan and Baht found that the TPC of unripe kundang (or Maprang; MP) fruit to be in the range of 0.023–0.050 mg GAE/mg MPFEs [9]. This is higher than that of the ripe fruit that were in the range of 0.012–0.020 mg GAE/mg MPFEs. Our results are similar to that of Thummajitsakul and Silprasit who reported that the TPC 95% etanolic leave extracts ranged from high to low levels, with mayong chid being the highest at 0.701 mg GAE/mg MPLEs, maprang prieyo at 0.680 mg GAE/mg MPLEs, and maprang wan at 0.531 mg GAE/mg MPLEs [28]. Andina and Musfirah found that the TPC was 0.137 mg GAE/mg MPCEs and 0.068 mg GAE/mg MPLEs for the ethanolic extraction of Ramania (Maprang) cortex and leaves, respectively [29]. All together, the highest total polyphenolic content of *Bouea macrophylla* Griffith was obtained from seed kernels followed by the cortex, leaves, and lastly the fruit itself.

### 3.3 Antioxidation properties of maprang (*Bouea macrophylla* Giffith) seed kernel extracts

The antioxidant activities of maprang seed kernel extracts were evaluated using TEAC, FRAP, and DPPH assays, and the results are shown in Table 1. The TEAC values of all three maprang varieties of both ripe and unripe fruit in all four ethanolic solvent systems exhibited antioxidant properties that varied between a minimum value of 1.34 ± 0.69 μg TE/μg MPSEs (MPWO25) and a maximum value of 3.17 ± 0.63 μg TE/μg MPSEs (MPWO75). These results indicate that the MPSEs have one to three times the antioxidation capacity than Trolox. No significant differences (*p* < 0.05) of mean values were found regarding the percentage of ethanol of all unripe fruit specimens and nearly all ripened fruit specimens, with the exception of maprang wan MPWO75 which showed significant differences from MPWO95 and MPWO25. We found no significant differences (*p* < 0.05) of mean values within unripened and ripened fruits of each maprang variety within the solvent extraction systems, except for MPWG75 and MPWO75 which were significantly different**.** Within the maprang variety the antioxidant capacity of the extracts obtained from maprang wan, maprang prieyo, and mayong chid were not significantly different (*p* < 0.05) among both the unripe and ripened fruit.

In order to determine the antioxidation properties of MPSEs by FRAP assay we obtained mean values that varied from 80.81 ± 12.56 μg Fe^2+^E/μg MPSEs (MPWO25) to 139.86 ± 18.36 μg Fe^2+^E/μg MPSEs (MPWO75). We found no significant differences (*p* < 0.05) in the mean values between the unripe and ripened fruit. There were only two significant differences evident among the maprang varieties, with the unripened fruit containing 95% ethanol (MPPG95-MYCG95) and the ripened fruit containing 25% ethanol (MPWO25-MYCO25). For maprang wan, the ethanol percentages in the ripened fruit MPWO50 (89.54 ± 11.67 μg Fe^2+^E/μg MPSEs) were significantly different from MPWO75 and MPWO25. For maprang prieyo, we found that only the unripened fruit MPPG25 (116.46 ± 17.93 μg Fe^2+^E/μg MPSEs) was significantly different to the other varieties, such as the MPPG50, MPPG75, and MPPG95. In the mayong chid, we found that only three pairs of the unripened fruit revealed significant differences; these being MYCG25-MYCG50, MYCG25-MYCG75, and MYCG50-MYCG75 based on the results of the Tukey's test (see Table 1).

We found no significant differences by DPPH assay *(p* < 0.05) in a comparison of any mean pairs within maprang varieties, maturation level of maprang fruit, or percentage of ethanol in any particular extraction system. These mean values (IC_50_) presented a minimum of 20.87 ± 1.90 μg/mL (MYCO95) and a maximum of 46.34 ± 22.64 μg/mL (MYCO95). Vitamin C, being the positive control IC_50_, was 13.80 ± 2.26 μg/mL. Nithitanakool et al determined that the IC_50_ of Vitamin C (2.20 ± 2.26 μg/mL) on DPPH radical scavenging was lower than in our results, and they also found that IC_50_ was 1.95 ± 0.04 μg/mL for mango seed kernel extracts [30]. Our results indicated that MPSEs exhibited 1.5–3.5 times less the DPPH radical scavenging than Vitamin C. Notably, Zainah et al reported that *Bouea macrophylla* Griffith seed extract by hot water extraction was highly active in scavenging DPPH radicals with IC_50_ 4.73 ± 0.51 μg/mL, and this activity was greater than among MPSEs [26]. Recently, it has been reported that maprang fruit extracts (both unripe and ripe) exhibit antioxidation properties [9]. It has been found that the highest activity of extracts was obtained from unripened fruit ranged from highest to lowest during extraction using methanol > ethanol > water. The anitioxidant values obtained by using FRAP, DPPH, and ABTS assays were 12.78 mM Fe (II)/100 g, 62.97% of DPPH inhibition and 99.17% of inhibition, respectively. However, the results from the unripened fruits, in particular the ABTS assay, revealed the same antioxidant values between the extracts obtained by methanol and by ethanol. Andina and Musfirah found that the ethanolic extraction of the maprang cortex and leaves scavenged DPPH radicals by IC_50_ at 20.03 μg/mL and 55.83 μg/mL, respectively [29]. Thummajitsakul and Silprasit reported that the ethanolic extracts obtained from the leaves of three maprang varities, maprang prieyo, mayong chid, and maprang wan, exhibited 50% ABTS^·+^ scavenging activity with (EC_50_) being 0.24 ± 0.08, 0.73 ± 0.07, and 0.70 ± 0.06, respectively [28]. In terms of the influence of the extraction solvents on antioxidant activity, Onivogui *et al.* found that the seed extracts of the *Anisophyllea laurina* R. Br. ex Sabine fruit revealed the highest activity in the ethanol solvent; followed by methanol, ethyl acetate, and water, respectively [31]. This was true for all three types of assays that involved the FRAP, DPPH, and ABTS assays. These antioxidant capacities correspond to the high presence of total phenolic content, total flavonoids, total tannins, and total anthocyanin contents that were found in the seed extracts.

### 3.4 Proximate and mineral content of maprang seed kernels obtained from the unripe and ripe fruit

Notably, this is the first time that the proximate composition of fresh seed kernels obtained from both unripe and ripe maprang fruit has been determined. These compositions are presented in Table 2. Most of the three maprang varieties contain carbohydrates and protein. In terms of the total dietary content, it was found that the seed kernels of the ripened fruit had higher levels of energy than the seed kernels of the unripened fruit. The unripe seed kernels contained 38–43 g/100 g carbohydrates, total dietary fibre of 5–7 g/100 g, 1.9–2.2 g/100 g of protein, moisture = 53–58 g/100 g, and energy = 168–187 kCal/100 g. The ripe seed kernels contained 47–55 g/100 g carbohydrates, total dietary fibre of 6–10 g/100 g, protein = 2.1–2.4 g/100 g, and energy = 205–239 kCal/100 g. Our results indicated that maprang seed kernels are a rich source of carbohydrates and dieatary fiber. Carbohydrate content was higher in the maprang seed kernels than in the mango seed kernels (ripe 32.8 g/100 g) [32] and for the maprang fruit (ripe 11.3 g/100 g) [8]. Notably, maprang fruit is higher in carbohydrate content even in the freeze-dried form (unripe 88.62 g/100 g; ripe 91.81g/100 g) [33], while the carbohydrate content of the mango kernel powder was 74.61 g/100 g [34]. The energy content is largely due to carbohydrate content followed by protein and fat contents. The dietary fiber content of the maprang seed kernels were higher than in the maprang fruit (ripe: 0.023 g/100 g wet; unripe: 6.39 g/100 g dry, ripe: 3.64 g/100 g dry) [8, 34] and mango seed kernels (2.0–3.2 g/100 g) [32,34] when analyzed through both wet and dry weight methods. Whereas, fat (0.8–0.9 g/100 g) and ash (1 g/100 g) contents were at the same level for both the unripe and ripe fruit in all three of the maprang varieties, yet they were less in the maprang seed kernels than in the mango seed kernels at 12.8 g/100 g fat and 2.0 g/100 g ash [32]. However, these figures were notably higher in the maprang seed kernels than in the maprang fruit (fat 0.02 g/100 g; ash 0.02 g/100 g) [8]. Maprang fruit in both fresh and freeze-dried forms presented protein content of 0.04 g/100g fresh and of 0.47–0.64 g/100 g in the dry form [8, 33]. Maprang fruit possesses less protein than maprang seed kernels (1.9–2.4 g/100 g), and was also lower in protein content than mango seed kernels (6–8 g/100 g) [32,34]. The moisture content showed decreasing levels from the unripened (53–58 g/100 g) to the ripened (40–49 g/100 g) forms of the fruit. The moisture content of the maprang seed kernels of the ripened fruit was similar to the levels found in the mango seed kernels (44.4 g/100 g; ripe) [32], and revealed about 50% less moisture content than the fresh maprang fruit (86 g/100 g) [8,33]. Mineral content was only determined in the ripe fruit with the most prevalent mineral being potassium (240–480 mg/100 g), followed by phosphorus (54–118 mg/100 g), magnesium (54–105 mg/100 g), calcium (16–34 mg/100 g), sodium (5–8 mg/100 g), zinc (5–6 mg/100 g), and iron (0.5–1 mg/100 g). Among the three maprang varieties, total mineral content revealed no sodium, and zinc was found to be at the highest level in the ripe mayong chid (MYCO), followed by ripe maprang wan (MPWO) and ripe maprang prieyo (MPPO) at an approximate ratio of 2:1.22:1, respectively. The average mineral content of maprang seed kernels (see Table 2) was lower than in the mango seed kernels as has been reported by Elegbede et al [32].

**Table 2:** Proximate compositions and mineral contents of maprang seed kernels.

Item	Unit	Maprang seed kernel extracts							
		Unripe (Green: G)				Ripe (Orange: O)			
		MPWG	MYCG	MPPG	Mean ± SD	MPWO	MYCO	MPPO	Mean ± SD
1. Ash	g/100g	0.98	0.98	1.03	1.00 **±** 0.03	0.96	0.98	0.98	0.97 **±** 0.01
2.Carbohydrate	g/100g	38.11	39.13	42.69	39.98 **±** 2.40	51.71	46.98	55.39	51.36 **±** 4.22
3.Dietary fiber (total)	g/100g	5.28	6.63	6.09	6.00 **±** 0.68	6.25	9.61	7.47	7.78 **±** 1.70
4.Energy	kCal/100g	168.32	172.67	187.20	176.07 **±** 9.89	223.71	205.10	238.65	222.49 **±** 16.81
5.Fat	g/100g	0.92	0.83	0.93	0.89 **±** 0.06	0.91	0.86	0.93	0.90 **±** 0.04
6.Moisture	g/100g	58.09	56.98	53.33	56.13 **±** 2.49	44.25	48.82	40.52	44.53 **±** 4.16
7.Protein (%Nx6.25)	g/100g	1.90	2.17	2.02	2.03 **±** 0.14	2.17	2.36	2.18	2.24 **±** 0.11
8.Calcium (Ca)	mg/100g	N/A	N/A	N/A	N/A	21.20	34.20	16.20	23.87 **±** 9.29
9.Iron (Fe)	mg/100g	N/A	N/A	N/A	N/A	0.77	1.04	0.53	0.78 **±** 0.26
10.Magnesium (Mg)	mg/100g	N/A	N/A	N/A	N/A	61.4	105.5	54.20	73.70 **±** 27.77
11.Phosphorus (P)	mg/100g	N/A	N/A	N/A	N/A	64.60	118.10	54.00	78.90 **±** 34.36
12.Potassium (K)	mg/100g	N/A	N/A	N/A	N/A	258.00	482.00	239.70	326.57 **±** 134.92
13.Sodium (Na)	mg/100g	N/A	N/A	N/A	N/A	5.926	6.279	8.159	6.79 **±** 1.20
14.Zinc (Zn)	mg/kg	N/A	N/A	N/A	N/A	5.11	6.40	5.00	5.50 **±** 0.78

N/A refers to no analysis

### 3.5 HPLC fingerprint and phytochemical content of maprang seed kernel extracts

**Fig. 2:**
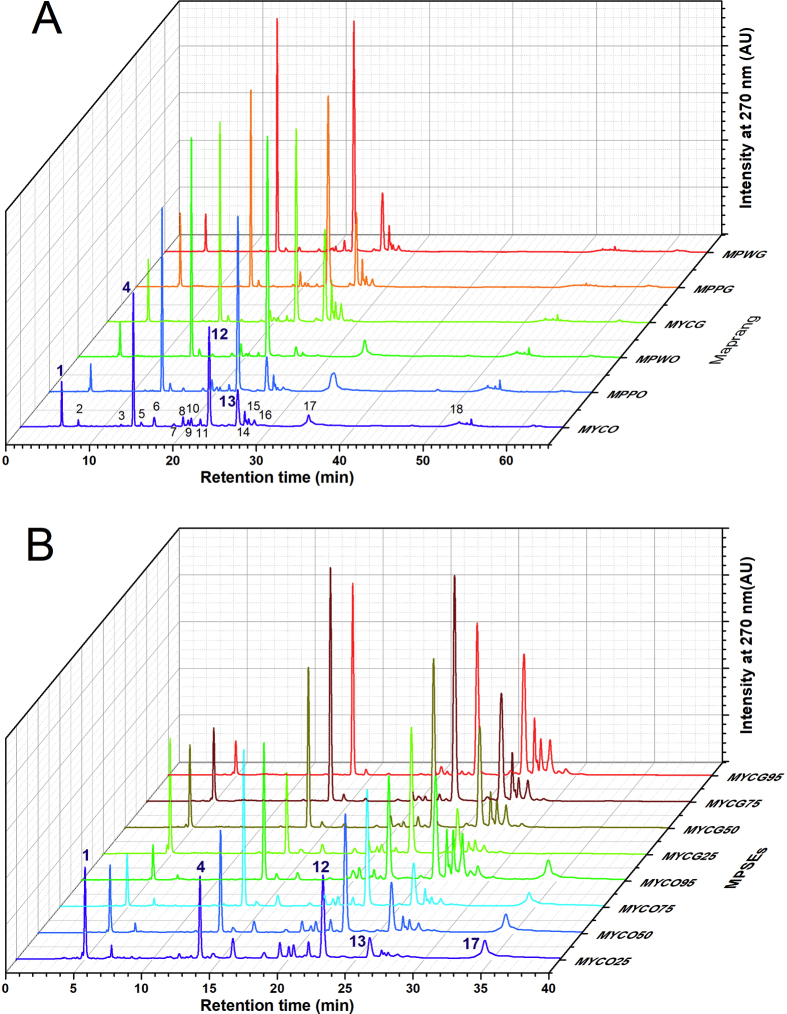
(A) HPLC fingerprints of maprang flavonoids obtained from maprang wan (MPWG & MPWO), maprang prieyo (MPPG & MPPO) and mayong chid (MYCG & MYCO). (B) HPLC fingerprints of maprang flavonoids obtained from green and ripe mayong chid seed extracts using various solvent extraction systems. MPSEs, Maprang seed extracts; G, Green (unripe fruit); O, Orange (ripe fruit); MPW, Maprang wan; MPP, Maprang prieyo; MYC, Mayong chid; MYCG25, MYCG50, MYCG75, and MYCG95: 25%, 50%, 75% and 95% Ethanolic seed extract of MYCG, respectively; and MYCO25, MYCO50, MYCO75, and MYCO95: 25%, 50%, 75% and 95% Ethanolic seed extract of MYCO, respectively.

**Fig. 3:**
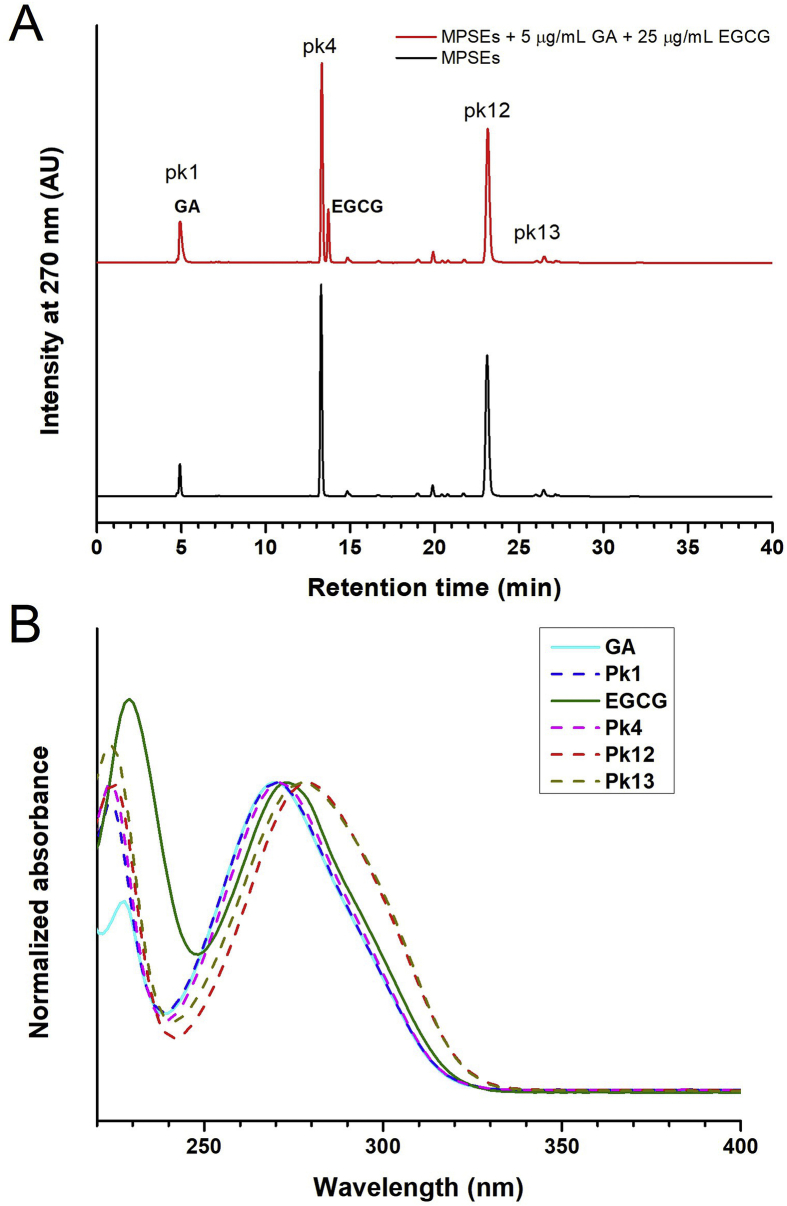
(A) HPLC chromatogram of only green maprang with the addition of standard GA and EGCG (B) UV-Vis spectral analysis of 4 major peaks of the maprang seed extracts.

The HPLC fingerprints at a wavelength of 270 nm for the maprang seed kernel extracts of three varities, maprang wan (MPW), maprang prieyo (MPP), and mayong chid (MYC), in both the unripe (G) and ripe (O) forms of the fruit were investigated and the results are presented in Fig. 2. As is shown in Fig. 2, all three of the maprang varieties displayed similar RP-HPLC chromatograms and contained 18 phytochemicals (**1**–**18**) being indicated as numeric labelled-peaks. Most of them (**1–16:** Pk1-Pk16) were presented at 30 minutes of retention time. In particular, the phytochemicals and the four major compounds of the maprang seed kernels were compounded at the peak numbers **1**, **4**, **12**, and **13**. Their concentrations were determined by the percentage of area in the peak as is shown in Table 3. The most prevalent compound was **12**, followed by **4,** and either **13** or **1** based on the extraction solvent used. The concentrations using 75% Ethanol as an extraction solvent gave the highest sum of the four peak areas in all maprang varieties by 78.4% (MPWG75), 78.68% (MPPG75), 72.89% (MYCG75), 73.43% (MPWO75), and 60.11% (MYCO75), and 62.94% in MPPO75 (64.8% MPPO95 highest). It is remarkable that **12** (24–40%) followed by **4** (19–26%) was the most abundant compound of maprang seeds in all three varieties for both the unripe and ripe fruit. HPLC chromatograms of each ethanolic solvent is represented in Fig. 2B. We observed that compound **17** was not only found in the unripe maprang fruit (MPWG, MPPG and MCYG), but that it also appeared later in the ripe maprang fruits (MPWO, MPPO and MCYO), as well. In addition, intensity of peaks **13**, **14**, **15** and **16** decreased in the ripened fruit specimens when compared to the unripe fruit specimens. It could be possible that these compounds (**13** to **17**) are related metabolites that form during the maturation process of the maprang fruit. Whereas, peak **18** might represent a highly polymerized form of gallotannins. We also found that compound **1** increased in concentration relative to an increase in water content, or by a decrease in ethanol in the extraction solvent (95% < 75% < 50% < 25). Compound **1** (*Rt* 5.05 minutes) and compound **11** (*Rt* 21.73 minutes) were considered to be gallic acid (GA) and ellagic acid (EA), respectively. Others, such as compound **4** (*Rt* 13.68 min), **12** (*Rt* 22.82 min)**, 13** (*Rt* 26.22 min), did not correspond to any standard polyphenol that was found in this study (see Fig. 3 and Table 4). Interestingly, compound **4** showed the same retention time as (-)-epigallocatechin gallate (EGCG) (*Rt* 13.68 minutes), but was not the identical compound, as is shown in Fig. 3A. By observing the spikes of GA and EGCG in the MPSEs solution, we obtained a splitting peak between compounds **4** and EGCG. The HPLC corresponding UV-Vis absoption spectral of these compounds, **1**, **4**, **12**, **13**, GA, and EGCG are shown in Fig. 3B. The maximum absorption peaks of compound **4** was 223 and 271 nm, whereas for the EGCG they were 229 and 273 nm. We observed a similar shape of the spectrum with very similar characteristic peak absorptions (λ1: 223–235 nm, λ2: 276–280 nm; shoulder: 295–300 nm) for the compound peaks **3**, **5**–**10**, **12**–**17**. These compounds possibly belong to catechins and/or hydrolysable tannins (gallotannins) based on their electronic absorption characteristics [35]. In the future, we intend to further study these characteristics and thereby identify these compounds. There are numerous research groups that have reported that gallic acid, ellagic acid, methyl gallate, catechins, and gallotannins are the phytochemicals found in the mango (*Mangifera indica* L.) seed kernels, and that they belong to the same Anacardicaeae family as the maprang fruit [30, 36, 37, 38]. It is well known that gallic acid, (+)-catechin and (-)-epicatechin, procyanidins, and galloyl compounds are abundant in grape (*Vitis vinifera* L.) seed extract [39]. Among the maprang seeds we harvested, there were no detected procyanidins by RP-HPLC-DAD. In addition, there were compounds present that also included flavonoids and glycosides that have been found in other fruit seed extracts [27], such as longan (*Dimocarpus longan* Lour) [14, 40], lichit (*Litchi chinensisSonnnerat, Sapindaceae*) [41, 42], and tamarind (*Tamarindus indica* L.) [13].

**Table 3:** Abundance of 4 major phytochemicals of MPSEs: Pk1 (*Rt* = 5.05 min), Pk4 (*Rt* = 13.68 min, Pk12 (*Rt* = 22.82 min) and Pk13 (*Rt* = 26.22 min).

MPSEs (Green) 300 μg/mL	Area (%)					MPSEs (Orange) 300 μg/mL	Area (%)				
	Pk1	Pk4	Pk12	Pk13	Sum of 4Pks		Pk1	Pk4	Pk12	Pk13	Sum of 4Pks
MPWG95	2.90	21.96	30.82	18.44	74.12	MPWO95	1.66	22.35	32.04	11.38	67.43
MPWG75	3.45	24.57	38.34	12.04	78.40	MPWO75	3.70	26.11	41.41	2.21	73.43
MPWG50	4.78	17.30	29.75	16.50	68.33	MPWO50	4.47	20.01	33.81	6.59	64.88
MPWG25	11.23	14.50	25.36	12.18	63.27	MPWO25	6.76	12.17	21.06	3.70	43.69
MPPG95	4.59	20.46	28.19	17.25	70.49	MPPO95	1.55	20.64	27.26	15.35	64.80
MPPG75	7.69	21.16	35.45	14.38	78.68	MPPO75	2.76	20.64	31.56	7.98	62.94
MPPG50	10.81	14.29	30.90	13.03	69.03	MPPO50	3.74	18.45	27.98	10.89	61.06
MPPG25	20.92	8.33	11.24	4.34	44.83	MPPO25	5.64	13.16	19.18	4.96	42.94
MYCG95	2.89	18.04	23.08	21.84	65.85	MYCO95	2.57	12.23	14.33	18.58	47.71
MYCG75	5.45	19.81	29.99	17.64	72.89	MYCO75	5.74	19.42	23.60	11.35	60.11
MYCG50	7.36	16.24	27.33	19.15	70.08	MYCO50	6.91	12.42	23.39	11.89	54.61
MYCG25	14.20	11.50	28.26	11.89	65.85	MYCO25	10.04	10.37	17.57	9.97	47.95

**Table 4:** Characterisitc UV-Vis absorption of 17 maprang polyphenols and standard polyphenols.

Selected standard polyphenols		Retention time (min)	Abs_max_ (nm)	MPSEs		Retention time (min)	Abs_max_ (nm)
No.	Name			Peak no.	Assigned		
1	Gallic acid (GA)	4.98	227, 270	1	Gallic acid	5.05	223, 270
2	Pyrocatechol (PyC)	6.82	225, 275	2	NA	6.97	259, 293
3	(-)-Gallocatechin (GC)	7.36	226, 270	3	NA	12.24	226, 276
4	Procyanidin B1	9.34	233, 278	4	NA	13.68	223, 271
5	(+)-Catechin (C)	10.31	230, 279	5	NA	14.64	225, 276
6	Caffeine (CF)	13.11	220, 265	6	NA	16.31	224, 280
7	(-)-Epigallocatechin gallate (EGCG)	13.68	229, 273	7	NA	18.70	224, 276
8	(-)-Epicatechin (EC)	13.91	230, 278	8	NA	19.67	224, 277
9	(-)-Gallocatechin gallate (GCG)	15.78	226, 273	9	NA	20.25	223, 277
10	*trans*-Ferulic acid (FA)	17.46	226, 321	10	NA	20.59	223, 278
11	(-)-Epicatechin gallate (ECG)	18.37	226, 272	11	Ellagic acid	21.73	253, 367
12	Ellagic acid (EA)	21.45	253, 367	12	NA	22.82	223, 279
13	Resveratrol (RV)	25.28	235, 310	13	NA	26.22	224, 277
14	Tannic acid (TA)	Multi peaks 26–35	274	14	NA	27.20	223, 278
15	Kaempferol (KF)	35.75	264, 364	15	NA	27.49	223, 277
16	Quercetin (QT)	30.24	254, 365	16	NA	28.18	223, 276
17	Apigenin (AN)	36.08	267, 336	17	NA	35.00	223, 276

NA is not assigned

### 3.6 Anticancer properties of maprang seed kernel extracts

Previous HPLC results indicate that the maprang seed extracts obtained from unripened fruit revealed higher levels of maprang polyphenols than the ripe fruit. Consequently, they have been selected for testing of their toxicity in doxorubicin-sensitive and -resistant leukemic (K562, K562/*adr*) and lung cancer (GLC4 and GLC4/*adr*) cells. Data regarding how unripe maprang seed kernel extracts can inhibit cancer cell growth by 50 percent (IC_50_) using various ethanolic extraction solvents is presented in Table 5. The concentrations of maprang seed extract that inhibited four cancer cell growth types by IC_50_ varied between 3 and 45 μg/mL. The maprang variety extracts that displayed the highest toxicity toward all four cancer cells was MPWG75 (IC_50_: 4–16 μg/mL), followed by MPPG50 (IC_50_: 2–5 μg/mL), and lastly MYCG75 (IC_50_: 6–16 μg/mL). To clarify, these concentrations were for maprang wan, maprang prieyo, and mayong chid, respectively. We found the IC_50_ of MPPG75 (2–14 μg/mL) to be at the same level as MPWG75 and MYCG75. All maprang seed extracts except MPWG75, MPWG95, and MYCG95 were more toxic (having less IC_50_ value) to doxorubicin-resistant cells (K562/*adr*, GLC4/*adr*) than to doxorubicin-sensitive cells (K562, GLC4), as the resistance factor was less than 1 (see Table 5). The RF values of doxorubicin were 72 and 16.5 for leukemic (K562, K562/*adr*) and lung cancer (GLC4 and GLC4/*adr*) cells [43]. In addition, maprang seed extracts showed greater toxicity against cancer cell growth in lung cancer cells than in leukemic cells. All together, our results suggest that maprang seed extracts obtained by 75% ethanol solvent had the greatest number of maprang polyphenol (compounds **4**, **12**, **13**, and **1**; see Table 5), and this was found to be the best extraction method for anticancer treatments. Notably, these results are consistent with those previously reported by Suttana et al [43]. We have tested the anticancer properties of maprang seed extracts by obtaining a serial extraction using chloroform, acetonitrile, ethanol, and water. We found that the ethanolic maprang seed extracts demonstrated the highest cytotoxicity to four cancer cell lines, K562, K562/*adr*, GLC4, and GLC4/*adr* with their IC_50_ values being equal to 8.9 ± 2.6, 5.8 ± 2.2, 10.9 ± 2.2, and 6.9 ± 1.0 μg/mL, respectively. Conversely, the IC_50_ of doxorubicin was found to be 0.01 ± 0.00, 0.72 ± 0.72, 0.02 ± 0.01, and 0.33 ± 0.21 μg/mL for K562, K562/*adr*, GLC4 and GLC4/*adr*, respectively. The screening antiprolifertive activity test results of *Bouae macrophylla* Griffith seed extracts on human squamous cell carcinoma (HTB-43), breast cancer (MCF7), and (MDA-MB-231) cell lines using MTT assay have already been reported [44]. It was found that aqueous, methanolic, chloroform, and hexane extracts exhibited promising inhibition activity against HTB43 cell lines with the IC_50_ values being 29.32 ± 5.80, 18.65 ± 2.94, 21.14 ± 6.97 and 34.36 ± 16.50 μg/mL, respectively. Only the hexane extractdisplayed inhibition against MCF7 (59.07 ± 5.76) and MDA-MB-231 (123.35 ± 28.65). Ethanolic seed extracts of mangos (*Mangifera indica*), which belong to the same Anacardiaceae family as the maprang fruit, exhibited anticancer properties against human breast cancer MCF-7 and MDA-MB-231 cells with IC_50_ values of 30 and 15 μg/mL, respectively. However, these extracts showed low toxicity to normal breast cancer MCF-10A cells [45] and showed hepatoprotective properties in rats with liver injuries induced by carbon tetrachloride (CCl_4_) [30].

**Table 5:** Cytotoxic activity of maprang seed extracts against human cancer cell lines (K562 and its P-gp over-expressed K562/*adr*, GLC4 and its MRP-1 over-expressed GLC4/*adr*).

Cell line	IC_50_ (μg/mL) Mean ± SD (n = 3)				Resistance Factor RF = (IC_50_ resistant cell/IC_50_ sensitive cell)			
	MPWG25	MPWG50	MPWG75	MPWG95	MPWG25	MPWG50	MPWG75	MPWG95
K562	25.22 ± 0.40	13.43 ± 1.08	15.57 ± 0.92	14.75 ± 0.25	-	-	-	-
K562/*adr*	21.19 ± 0.53	13.88 ± 0.43	10.87 ± 0.22	14.75 ± 0.38	0.84	1.03	0.70	1
GLC4	16.90 ± 0.30	9.60 ± 0.57	4.43 ± 0.39	3.56 ± 0.25	-	-	-	-
GLC4/*adr*	4.84 ± 0.14	8.31 ± 0.25	8.72 ± 0.84	8.30 ± 0.28	0.28	0.86	1.97	2.33
	MPPG25	MPPG50	MPPG75	MPPG95	MPPG25	MPPG50	MPPG75	MPPG95
K562	16.90 ± 0.41	5.00 ± 0.19	13.5 ± 0.33	36.00 ± 0.74	-	-	-	-
K562/*adr*	16.00 ± 0.57	2.70 ± 0.15	9.40 ± 0.20	18.80 ± 0.48	0.95	0.54	0.70	0.52
GLC4	12.90 ± 0.62	2.70 ± 0.18	6.80 ± 0.13	15.50 ± 0.23	-	-	-	-
GLC4/*adr*	6.00 ± 1.07	1.96 ± 0.15	2.70 ± 0.25	11.32 ± 0.20	0.46	0.73	0.40	0.73
	MYCG25	MYCG50	MYCG75	MYCG95	MYCG25	MYCG50	MYCG75	MYCG95
K562	43.90 ± 1.33	35.34 ± 0.80	16.43 ± 0.98	13.42 ± 0.29	-	-	-	-
K562/*adr*	27.62 ± 1.00	26.30 ± 0.52	11.28 ± 0.42	13.00 ± 0.10	0.63	0.74	0.68	0.97
GLC4	23.33 ± 0.65	10.46 ± 0.86	10.45 ± 0.22	3.56 ± 0.10	-	-	-	-
GLC4/*adr*	8.30 ± 0.17	10.00 ± 0.58	6.16 ± 0.15	8.30 ± 0.18	0.35	0.96	0.59	2.33

### 3.7 Antimicrobial properties of maprang seed extracts

This study represents the first time that the antimicrobial properties of maprang seed extracts *in vitro* have been analyzed. Antimicrobial activity of maprang seed extracts in both unripe (MYCG95) and ripe (MYCO95) fruit specimens was pre-screened in *E. coli* using an agar well diffusion assay, and the results indicated a clear zone of inhibition taking place in both MYCG95 and MYCO95 with an average diameter of 21 and 19 mm, respectively. A broth microdilution method was performed and this process indicated that both MYCG95 and MYCO95 had an inhibitory effect against pathogenic gram-positive, gram-negative bacteria (15 strains), and yeast (C. *albican*) with the MICs ranging from 39.0-625.0 μg/mL. Notably, *V. parahemolyticus, S. boydii* and vancomycin resistant *E. faecalis* strains were particularly inhibited by ripe maprang seed kernel extracts with MICs at 52.0 ± 22.6, 78.1 ± 0 and 78.1 ± 0 μg/mL, respectively. This was the case for ripened seed extracts, but was also found to be the same for the unripened seed extracts. Notably, they also displayed active inhibition against *V. parahemolyticus* (MIC 39.0 ± 0 μg/mL)*, S. boydii* (MIC 104.1 ± 45.1 μg/mL)*,* and vancomycin resistant *E. faecalis* (MIC 104.1 ± 45.1 μg/mL). The degree of growth inhibition of drug resistant bacteria including methicillin resistant *S. Aureus* (MRSA; MICs: unripe 104.1 μg/mL, ripe 130.2 μg/mL), vancomycin resistant *E. Faecalis* (MICs: unripe 104.1 μg/mL, ripe 78.1 μg/mL) and ESBL producing *E. coli* (MICs: unripe 520.8 μg/mL, ripe 625.0 μg/mL) was observed by these seed extracts. Surprisingly, most of the microorganisms tested could be inhibited, but were unable to be killed with the MBC/MFC values being over 2,500 μg/mL. The MIC and MBC/MFC values of the maprang seed extracts against several microorganisms are summarized in Table 6. Maprang (*Bouea macrophylla* Griffith) seed kernels exhibited similar antimicrobial properties to mango (*Magnifera indica* L.) seed kernels. Again, they both belong to the Anacardiaceae family and their properties have been well documented. Kabuki et al reported that mango seed kernel extracts (MSKE) that were obtained using ethnanolic solvents exhibited antimicrobial activity against 43 strains (18 species) in both gram-positive and gram-negative bacteria. The MSKE showed remarkable antimicrobial activity against gram-positive strains that included *S. aureus*, *Bacillis* sp., *Clostidium* sp., and *Listeria monocytogenes* with the MICs being in a range of 50–500 μg/mL [46]. In addition, the MSKE was also found to be active against *Campylbacter jejuni* and *Yersinia enterocolitica* with the MICs for both specimens equaling 100 μg/mL. Jiamboonsri et al also reported that the ethanolic extraction obtained from the Thai mango “fahlun” seed kernel extract showed antimicrobial activity against *S. aureus* (ATCC 25923) and 19 clinically isolated MRSA strains. Both of these strains MICs were 470 μg/mL and the MBCs varied from 940 to 3,750 μg/mL [38]. It appears that the maprang seed kernel extracts obtained by us were more highly active in terms of antimicrobial activity than the mango seed kernel extracts were against methicillin resistant *S. Aureus*. In addition, Mutua et al reported that the MSKE methanolic extraction of four mango varieties of Kenya exhibited antimicrobial activity against *E. coli*, *S. aureus*, and *C. albican* based on an agar disk diffusion assay [34]. It is well known that the antimicrobial activity of plant extracts, including mango seed kernel extracts, is exerted by hydrolizable tanins or gallotannins with 4–9 galloyl groups, flavonoids, and phenolic acids. Pentaglloylglucopyranose is a major compound found in mango seed kernels [34, 37, 38]. Notably, the antimicrobial activity of gallotannins directly increased along with their molecular weight [47]. Moreover, the other plants belonging to the Anacardiaceae family also displayed antimicrobial activity. Gallotannins obtained from *Gall chinesis* exhibited antimicrobial activity against both gram-positive (*S. aureu, B. subtilis* and *B. cereus*) and gram-negative (*E. coli, S. typhimurium* and *S. Dysenteriae*) specimens with the MICs varying between 250 and 1,500 μg/mL [47]. Additionally, the extracts obtained from *Schinopsis brasiliensis* Engl. exhibited antimicrobial activity against both gram-positive and gram-negative bacteria with the MICs ranging from 390 to 1,250 μg/mL [48].

**Table 6:** **Antimicrobial activity of maprang seed extracts against several microorganisms**.

Microorganisms	Gram	MIC (μg/mL) Mean ± SD (n = 3)		MBC/MFC (μg/mL) Mean ± SD (n = 3)	
		MYCG95	MYCO95	MYCG95	MYCO95
*S. aureus* ATCC 25923	+	156.2 ± 0	156.2 ± 0	312.5 ± 0	312.5 ± 0
methicillin resistant *S. aureus* ATCC 43300	+	104.1 ± 45.1	130.2 ± 45.1	>2500	>2500
*E. faecalis*	+	312.5 ± 0	312.5 ± 0	>2500	>2500
vancomycin resistant *E. faecalis*	+	104.1 ± 45.1	78.1 ± 0	>2500	>2500
*E. coli* ATCC 25922	+	312.5 ± 0	312.5 ± 0	>2500	>2500
ESBL^1^ producing *E. coli*	-	520.8 ± 180.4	625 ± 0	>2500	>2500
*S. enteritidis*	-	625 ± 0	520.8 ± 180.4	>2500	>2500
*S. boydii*	-	104.1 ± 45.1	78.1 ± 0	>2500	>2500
*P. mirabilis*	-	520.8 ± 180.4	625 ± 0	2500	1250
*K. pneumoniae*	-	520.8 ± 180.4	625 ± 0	>2500	>2500
*E. aerogenes*	-	520.8 ± 180.4	312.5 ± 0	>2500	>2500
*V. cholera*	-	260.4 ± 90.2	156.2 ± 0	312.5 ± 0	312.5 ± 0
*V. parahemolyticus*	-	39.0 ± 0	52.0 ± 22.6	156.2 ± 0	208.2 ± 90.4
*P. aeroginosa*	-	312.5 ± 0	312.5 ± 0	>2500	>2500
*A. buamannii*	-	625 ± 0	625 ± 0	>2500	>2500
*C. albicans* ATCC 90028		208.3 ± 90.2	104.1 ± 45.1	>2500	>2500

1 ESBL = Extended-spectrum beta-lactamase.

## 4 Conclusion

Our results indicate that maprang (*Bouea macrophylla* Griffith) seed kernels are a new natural source of polyphenols, and that these polyphenols are more abundant than in any of the other parts of the maprang plant including the leaves, fruit, and the cortex of the fruit. Seed kernels also contain carbohydrates and dietary fiber. The mineral content includes potassium, phosphorus, magnesium, and calcium. Surprisingly, maprang seed extracts demonstrate greater anticancer and antibacterial activity in drug resistant cells than in drug sensitive cells. These results suggest that maprang seed polyphenols might have great potential in overcoming the multidrug resistance that typically occurs with regard to cancer and other harmful microorganisms. In conclusion, it should be noted that maprang (*Bouea macrophylla* Griffith) seeds are also a viable natural source of nutrition. The minerals and phytochemicals found in these seeds demonstrate bioactivity against cancer and bacteria, and there may be further applications for these seeds as a food source in addition to their use in medicines. This in-depth study may help to promote the cultivation of maprang trees in Thailand and help find other new natural sources of antioxidants that can be obtained from similar fruit by-products, which can potentially be useful in terms of health promotion among the general population and economic development.

## Declarations

### Author contribution statement

Nathupakorn Dechsupa: Conceived and designed the experiments; Performed the experiments; Analyzed and interpreted the data; Contributed reagents, materials, analysis tools or data; Wrote the paper.

Montree Tungjai: Performed the experiments; Analyzed and interpreted the data.

Jiraporn Kantapan, Sorasak Intorasoot: Performed the experiments; Analyzed and interpreted the data; Contributed reagents, materials, analysis tools or data; Wrote the paper.

### Funding statement

This work was supported by the Thailand Research Fund, Thailand (Grant number RDG5750047).

### Competing interest statement

The authors declare no conflict of interest.

### Additional information

No additional information is available for this paper.
